# Island biogeography of soil bacteria and fungi: similar patterns, but different mechanisms

**DOI:** 10.1038/s41396-020-0657-8

**Published:** 2020-04-27

**Authors:** Shao-peng Li, Pandeng Wang, Yongjian Chen, Maxwell C. Wilson, Xian Yang, Chao Ma, Jianbo Lu, Xiao-yong Chen, Jianguo Wu, Wen-sheng Shu, Lin Jiang

**Affiliations:** 1 School of Biological Sciences, Georgia Institute of Technology, Atlanta, GA 30332 USA; 2 Zhejiang Tiantong Forest Ecosystem National Observation and Research Station, School of Ecological and Environmental Sciences, East China Normal University, Shanghai, 200241 China; 3 Institute of Eco-Chongming (IEC), Shanghai, 202162 China; 4 School of Life Sciences & School of Ecology, Sun Yat-sen University, Guangzhou, 510275 China; 5 School of Life Sciences & School of Sustainability, Arizona State University, Tempe, AZ 85287 USA; 6 Anhui Province Key Lab of Farmland Ecological Conservation and Pollution Prevention, School of Resources and Enviro\nment, Anhui Agricultural University, Hefei, 230036 China; 7 College of Life and Environmental Sciences, Hangzhou Normal University, Hangzhou, 310036 China; 8 Shanghai Institute of Pollution Control and Ecological Security, Shanghai, 200092 China; 9 School of Life Sciences, South China Normal University, Guangzhou, 510631 China

**Keywords:** Community ecology, Microbial ecology, Biogeography

## Abstract

Microbes, similar to plants and animals, exhibit biogeographic patterns. However, in contrast with the considerable knowledge on the island biogeography of higher organisms, we know little about the distribution of microorganisms within and among islands. Here, we explored insular soil bacterial and fungal biogeography and underlying mechanisms, using soil microbiota from a group of land-bridge islands as a model system. Similar to island species-area relationships observed for many macroorganisms, both island-scale bacterial and fungal diversity increased with island area; neither diversity, however, was affected by island isolation. By contrast, bacterial and fungal communities exhibited strikingly different assembly patterns within islands. The loss of bacterial diversity on smaller islands was driven primarily by the systematic decline of diversity within samples, whereas the loss of fungal diversity on smaller islands was driven primarily by the homogenization of community composition among samples. Lower soil moisture limited within-sample bacterial diversity, whereas smaller spatial distances among samples restricted among-sample fungal diversity, on smaller islands. These results indicate that among-island differences in habitat quality generate the bacterial island species-area relationship, whereas within-island dispersal limitation generates the fungal island species-area relationship. Together, our study suggests that different mechanisms underlie similar island biogeography patterns of soil bacteria and fungi.

## Introduction

For centuries, islands have served as useful natural laboratories for addressing fundamental ecological and evolutionary questions [1–3]. The study of islands inspired the development of the theory of island biogeography [4], and, subsequently, a large body of empirical work on the biogeography of insular plants and animals [5–8]. However, although a growing body of research shows that microorganisms have similar biogeography as higher organisms [9–14], little is known about the biogeography of island-dwelling microbes [15]. Our knowledge on microbial biogeography is almost entirely derived from studies of continuous landscapes [9, 12, 14, 16–18] or virtual islands, such as bacteria in water-filled treeholes [19], ectomycorrhizal fungi on host plants [20, 21], microfungi in floral nectar [22], and diatoms in boreal springs [23]. However, islands differ from mainland systems in various aspects, including abiotic environmental conditions, the size of supported populations, and the extent of species dispersal, making it difficult to directly apply assembly mechanisms of communities on mainland to islands [24, 25]. Likewise, virtual islands, which are often substantially smaller than actual islands, may not necessarily replicate community assembly processes on actual islands [26, 27]. Therefore, there is a critical need to elucidate the biogeography and underlying assembly mechanisms of microbial communities for true island systems.

Understanding processes driving island biogeographical patterns has proven difficult. One reason for this difficulty is the failure to appreciate the broad spectrum of plausible mechanisms. Three main candidate mechanisms are often thought to contribute to positive island species-area relationships. Larger islands could harbor more species simply because of their ability to support more individuals (the sampling effect [28]). Higher species colonization rate and lower extinction rate may confer greater species diversity on larger islands (the area per se effect [28]), as predicted by the equilibrium theory of island biogeography [4]. Larger islands may also support more diverse species assemblages because they tend to contain a greater diversity of habitats (the habitat heterogeneity effect [28, 29]). However, besides these mechanisms, several other factors may also play important, but underappreciated, roles in producing species-area patterns. For example, smaller islands tend to contain disproportionally more edge habitats that experience greater levels of abiotic stress (e.g., wind turbulence, desiccation), which could result in overall lower habitat quality, and in turn, lower species diversity on smaller islands [30, 31]. Dispersal limitation, known to occur for microbial communities [13, 21], may not only shape communities across islands but also across localities within islands, especially for large islands where there are substantial distances among localities. Greater dispersal limitation among localities within larger islands may thus have the potential to contribute to island species-area relationships.

**Fig. 1:**
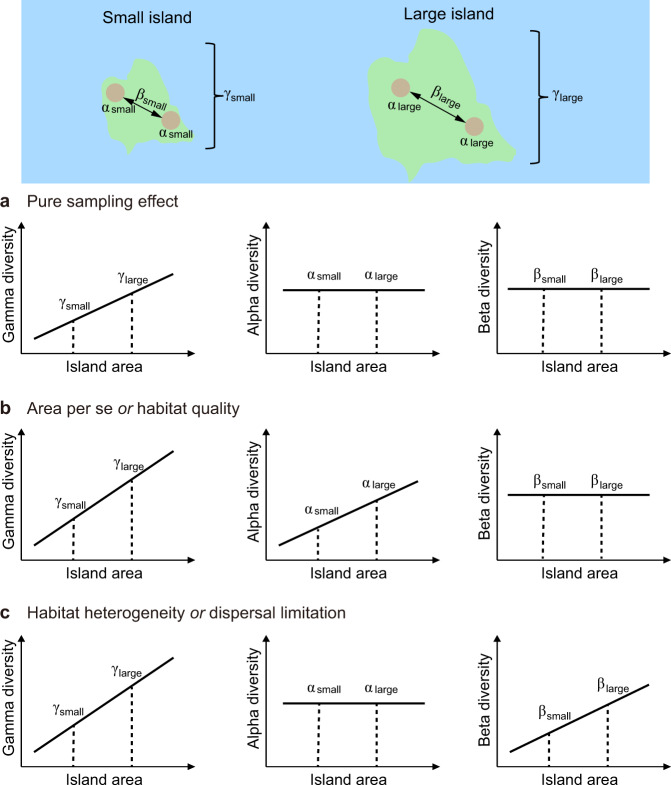
A conceptual diagram illustrating the potential mechanisms behind the positive island species-area relationships, assuming that all islands share the same regional species pool. The total number of species on an island (gamma diversity) is decomposed to species richness within samples (alpha diversity) and sample-to-sample species turnover (beta diversity) within the island. **a** Gamma diversity, but not alpha or beta diversity, increases with island area. This pattern is in line with the prediction of the sampling effect, which posits that larger islands contain more individuals and thus accumulate more species by chance. **b** Gamma and alpha diversity, but not beta diversity, increase with island area. This pattern is often considered as the evidence for the area *per se* effect, which emphasizes the importance of reduced local extinction risk on larger islands. Alternatively, we suggest that this pattern could also be explained by local habitat quality favoring more species on larger islands. **c** Gamma and beta diversity, but not alpha diversity, increase with island area. This pattern has often been explained by greater habitat heterogeneity on larger islands. Alternatively, we suggest that this pattern could also arise from stronger dispersal limitation on larger islands.

Here, we first propose a novel framework that helps to gain a more complete understanding of mechanisms driving insular species-area relationships. We then apply this framework to the islands of the Thousand-Island Lake in subtropical China to explore mechanisms driving soil bacterial and fungal species-area relationships on these islands. Building on previous work [28, 32, 33], this framework decomposes island-level diversity (henceforth gamma diversity) into species richness within samples (henceforth alpha diversity) and species turnover among samples (henceforth beta diversity) within islands (Fig. 1). Unlike previous work, our approach simultaneously considers common candidate mechanisms contributing to species-area relationships, as well as previously underappreciated mechanisms including difference in habitat quality among islands and dispersal limitation within islands. For example, the increased gamma diversity on larger islands, in the absence of alpha and beta diversity relationships with area, indicative of the negligible influence of the balancing between species colonization and extinction, habitat differences, or dispersal limitation on diversity within islands, would provide evidence for the importance of the sampling effect (Fig. 1a). A positive relationship between alpha, but not beta diversity, and island area would point to the importance of the area per se effect [28], as predicted by the equilibrium theory of island biogeography [4], or to improved environmental conditions favoring higher species diversity on larger islands (the habitat quality effect; Fig. 1b; [34]). On the other hand, a positive relationship between beta, but not alpha diversity, and island area could be attributed to greater habitat heterogeneity allowing the coexistence of species with different habitat requirements on larger islands (the habitat heterogeneity effect, Fig. 1c; [32, 33]). Alternatively, higher beta diversity within larger islands could simply arise from stronger dispersal limitation among localities that are spaced further apart (the dispersal limitation effect, Fig. 1c; [35]). By examining how both environmental and spatial factors influence alpha and beta diversity within islands, which has not been done before, this framework provides a useful approach for distinguishing ecological mechanisms generating island biogeographical patterns.

We applied this framework to study spatial patterns of soil bacterial and fungal diversity and test for their underlying mechanisms, using a cluster of 29 land-bridge islands in the Thousand Island Lake (TIL) in subtropical China as a model system. The 29 islands are of the same age, differ substantially in size (Fig. S1), and have experienced minimum levels of human disturbance since their formation, providing an excellent opportunity for exploring island biogeography questions. We collected a total of 306 soil samples from the 29 islands as well as the nearby mainland, and estimated soil bacterial and fungal composition and diversity via high-throughput sequencing. By simultaneously considering gamma, alpha, and beta diversity of soil bacteria and fungi within islands, as well as the relative importance of environmental and spatial factors for these diversity components, we aimed to unravel mechanisms underlying the biogeography of soil microbes on these islands.

## Methods

### Study sites and sampling

Our study was conducted in the Thousand Island Lake (29°22′N to 29°50′N and 118°34′E to 119°15′E), a man-made reservoir created by damming in 1959, in subtropical China. The flooding inundated an area of ~580 km^2^, transforming former hilltops in the area into islands. Islands are covered by secondary forests dominated by *Pinus massoniana* [36].

A cluster of 29 islands with minimum levels of human disturbance was selected as our study sites (Fig. S1). The size of the 29 islands varies from 0.08 to 1153.87 ha. We used a hierarchical sampling regime to collect soil samples. On each island, we established one to six permanently marked 20 × 20 m^2^ plots (two 10 × 10 m^2^ quadrats on the smallest island), with the number of plots roughly proportional to island area on the logarithmic (log_10_) scale [32]. Each plot was further divided into four 10 × 10 m^2^ quadrats. In addition, we set up one 10,000 m^2^ control plot on the adjacent mainland, with 20 quadrats (10 × 10 m^2^) evenly distributed within the plot. For each quadrat on the islands and mainland, four evenly distributed soil cores (3 cm diameter to 10 cm depth) were taken and mixed to form one composite sample, resulting in a total of 306 soil samples.

Sampling was carried out in mid-May 2015. Samples were placed in sterile plastic bags, sealed and placed on ice, and immediately transported to the laboratory. All soil samples were carefully homogenized prior to further treatment. For each sample, a ~2 g subsample was placed into a sterilized tube at −80 °C before DNA extraction. A second subset of fresh soil was used for soil moisture measurement, and a third subset of air-dried soil was preserved for subsequent soil chemistry analysis.

### Microbial analyses

Soil DNA was extracted by the MoBio PowerSoil DNA extraction kit (MO BIO Laboratories, Carlsbad, CA, USA). For bacteria, we targeted the V4 region of the 16 S ribosomal RNA (rRNA) gene, using 515-F (GTGCCAGCMGCCGCGGTAA) and 806-R (GGACTACHVGGGTWTCTAAT) primer pairs [37]. For fungi, we targeted the second nuclear ribosomal internal transcribed spacer (ITS2) region of the rRNA operon, using the ITS3 (GCATCGATGAAGAACGCAGC) and ITS4 (TCCTCCGCTTATTGATATGC) primer pairs [38]. To permit multiplexing of samples, a 12-bp barcode unique to each sample was added to the reverse primers during primer synthesis. PCR amplification was performed in triplicate for both the 16 S rRNA gene and the ITS2 region with 20 μl reactions containing 10 μl 2 × premix (TaKaRa Bio, Mountain view, CA, USA), 0.4 μl of both forward and reversed primers (10 mM), and 10 ng template DNA. The PCR program for 16 s rRNA gene was as follows: preheat 1 min at 94 °C, then 30 cycles of 94 °C for 10 s, 53 °C for 25 s, 68 °C for 45 s, and a final extension at 68 °C for 8 min. As for ITS2 region, the PCR program was as follows: preheat 15 min at 95 °C, then 30 cycles at 95 °C for 10 s, 55 °C for 30 s, 72 °C for 1 min, and a final extension at 72 °C for 10 min. Negative controls were included in each batch of DNA extraction and PCR. PCR products from all samples were pooled together in equimolar concentrations, and purified by using the E.Z.N.A.® Gel Extraction Kit (Omega Bio-tek, Norcross, GA, USA). The pooled PCR products were subsequently sequenced on a 2 × 300 bp paired-end Illumina MiSeq platform (Illumina; San Diego, CA, USA) at Sun Yat-sen University (Guangzhou, China).

The raw sequence data were processed with the Mothur software package [39] for quality filtering and assembling of pair-end reads. Strict quality control steps were applied to the sequencing data. First, assembled contigs without exact match to one of the barcode sets or primers (degenerate bases were not taken into consideration) were discarded. Subsequently, the remaining sequences were clustered into operational taxonomic units (OTUs) based on a 97% similarity threshold with the UPARSE algorithm [40], using the “-cluster_otus” command in USEARCH, with chimera sequences identified and eliminated during the procedure. Sequences found only once across all samples were treated as singletons and removed from subsequent analyses. Taxonomic classification of each OTU of bacteria and fungi was determined using the Ribosomal Database Project (RDP) classifier [41] with a confidence threshold of 0.8 (bacteria) and 0.5 (fungi) against the SILVA [42] and the UNITE [43] database, respectively. OTUs that were not classified into bacteria or fungi were removed. The samples were rarefied to an even number of sequences per sample (8381 and 2720 for bacteria and fungi, respectively).

### Soil properties

We measured nine soil chemical properties that could potentially influence microbial communities (Table S1). Soil moisture was measured by oven drying 10 g of fresh soil at 105 °C until constant weight. Soil pH was measured in soil suspension with a 1:2.5 ratio of soil to deionized water. Soil total organic carbon (TOC) was measured by the potassium dichromate oxidation method following the modified Walkley-Black procedure, and total N was measured by the semimicro-Kjeldahl method (Kjeltec 2200 Auto Distillation Unit, FOSS, Hillerød, Sweden). Soil total P and available P were measured by the colorimetric method using a UV-Visible Spectrophotometer (UV-2550, Shimadzu, Kyoto, Japan). Soil available Ca, Mg, and Al were extracted by the Mehlich-III solution and measured using Inductively Coupled Plasma Optical Emission Spectrometry (Optima 2100 DV, Perkin-Elmer, Waltham, MA, USA). Prior to analysis, data on these soil properties were standardized to have a mean value of 0 and variance of 1.

### Statistical analyses

We assessed three components of bacterial and fungal diversity for each island: gamma, alpha, and beta diversity. Gamma diversity, which was defined as the estimated total richness of the whole island, was calculated as the Chao2 estimator based on OTUs’ incidence frequencies on each island [44]. Alpha diversity was defined as the average OTU richness per sample within an island. Beta diversity was defined as the average Bray–Curtis dissimilarity values among samples within the island, and visualized via non-metric multidimensional scaling (NMDS). We also calculated other diversity indices for gamma (i.e., Chao1, ACE, ICE, Jack1, Jack2), alpha (i.e., the exponential of Shannon entropy, the inverse Simpson index), and beta (i.e., Jaccard index, Sorenson index, the Bray–Curtis based Raup–Crick index, and measures based on Hill numbers) diversity [45, 46]. They all yielded similar results (Tables S2, S3). Further, to assess the potential influences of the sampling effect, gamma diversity was also calculated as the total OTU richness rarefied to an equal number of reads, or an equal number of samples, per island. We also calculated the rarefaction curve of each island with gamma diversity as a function of the sample size for each island. To compare within-island beta diversity across samples of similar spatial distances, we calculated the average beta diversity among samples within the 20 m × 20 m plots for each island. Island isolation was measured as the distance to the nearest island (DNI) or nearest distance to the mainland (NDM). We used linear and segmented regressions to assess the effects of island area and isolation on the mean alpha, beta and gamma diversity of soil bacteria and fungi on each island. Where appropriate, data were log transformed to improve model fit and improve the homoscedasticity of residuals.

We used multiple linear regressions and multiple regressions on distance matrices [47] to assess the importance of spatial and individual environmental factors on alpha and beta diversity, respectively. For alpha diversity, the nine soil properties of each soil sample were considered as independent variables. For beta diversity, we considered spatial distance among samples, and the dissimilarity in soil properties (based on Euclidean distances) within island as independent variables. The importance of the variables, as well as the variance explained by the models, were estimated using the *lmg* function in the R package *relaimpo* [48]. We then used variation partitioning to distinguish the contribution of spatial and environmental factors to beta diversity, where spatial distance and environmental dissimilarity among samples within island were used as predictor variables. Variable selection was implemented by using full subset model selection and by minimizing the Akaike Information Criterion (AIC), which alleviates variable collinearity [49]. Variation partitioning was performed with the *varpart* function in the R package *vegan* [50].

We further used structural equation modeling (SEM) to disentangle the causal pathways through which island spatial and environmental factors influence alpha, beta, and gamma diversity of soil bacteria and fungi. We constructed the same a priori model for soil bacteria and fungi, considering all possible mechanisms whereby island and soil characteristics influence microbial diversity, including the sampling effect represented by a direct link between island area and gamma diversity (i.e., island area influences gamma diversity without changing alpha and beta diversity; Fig. 1a). Habitat heterogeneity of each island was calculated as the average pairwise Euclidean distances among samples, based on the nine soil properties. We simplified the initial models by eliminating non-significant pathways before we attained the final models. Model adequacy was determined using the χ^2^ test and AIC. Structural equation modeling was conducted with the R package *sem* [51]. We performed all statistical analyses in R version 3.3.2 [52].

## Results

A total of 9,022,457 and 4,535,687 high-quality sequences were obtained for soil bacteria and fungi across all samples, respectively, with an average of 29,485 ± 10,109 (bacteria, mean ± SD) and 14,822 ± 9553 (fungi, mean ± SD) sequences detected per sample. After clustering sequences at the 97% similarity level and removing singletons, we obtained 20,078 and 10,579 operational taxonomic units (OTUs) for bacteria and fungi, respectively. After rarefying all samples to the same number of sequences (8381 for bacteria, 2720 for fungi), an average of 1434 ± 177 bacterial OTUs and 337 ± 52 fungal OTUs were detected per sample. On average, 4718 bacterial OTUs and 1287 fungal OTUs were detected per island, with the range being 1750–7788 for bacteria and 463 to 2229 for fungi. At the phylum level, soil bacterial communities were dominated by Proteobacteria (20.9–29.8%), Acidobacteria (16.1–30.6%), Verrucomicrobia (8.7–28.9%), and Chloroflexi (6.6–21.2%) across all islands (Fig. S2a). For fungal communities, Ascomycota (29.2–66.6%) and Basidiomycota (16.7–46.9%) were the dominant phyla (Fig. S2b), with Russulaceae (1.4–20.6%), Mortierellaceae (1.7–17.2%), Umbelopsidaceae (1.5–19.4 %), and Trimorphomycetaceae (1.1–14.1%) being the dominant families.

**Fig. 2:**
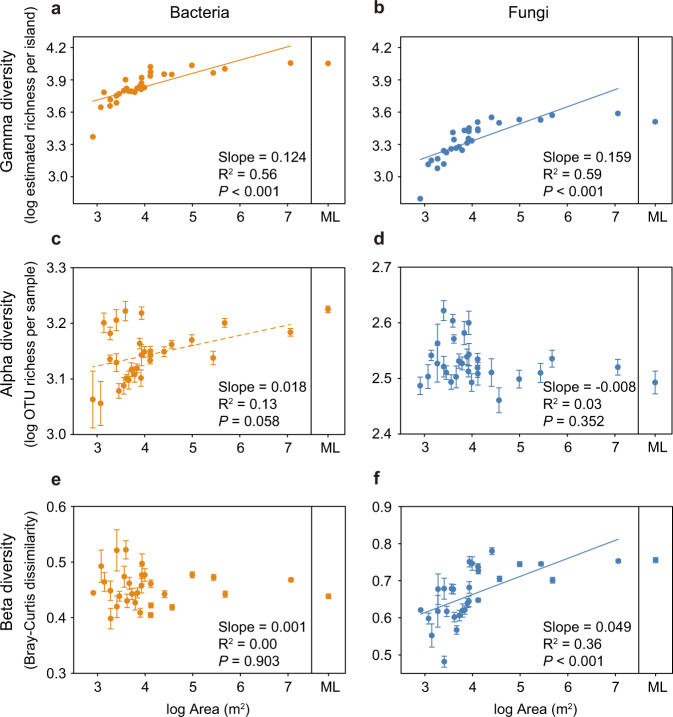
The effects of island area on the gamma, alpha and beta diversity of soil bacteria and fungi. Panels **a**, **c**, **e** are for bacterial gamma, alpha and beta diversity, respectively; panels **b**, **d**, **f** are for fungal gamma, alpha and beta diversity, respectively. Gamma diversity was measured as the estimated total OTU richness (based on Chao2 estimator) per island. Alpha diversity was measured as the average OTU richness per sample within each island. Beta diversity was measured as average pairwise Bray–Curtis dissimilarities among samples within each island. Error bars represent standard error. The solid lines represent significant linear regressions (*P* < 0.05) and the dashed lines represent marginally significant regressions (*P* < 0.10). ML indicates data from the mainland.

Both linear and segmented regressions showed that bacterial and fungal diversity at the island scale (gamma diversity) increased significantly with island area (Fig. 2a, b and Fig. S3). The gamma diversity-area relationships were less positive but remained statistically significant after we controlled for the variation in the total number of sequences (Fig. S4), or the total number of samples (Fig. S5), across islands, suggesting that the sampling effect played an important, but non-exclusive, role in driving the observed relationships. In addition, all islands shared similar-shaped rarefaction curves for gamma diversity, with larger islands generally showing greater gamma diversity at equal sample size (Fig. S6). The nearby mainland exhibited greater bacterial and fungal gamma diversity than all but the largest islands (Fig. 2a, b). Island isolation, measured as either DNI or NDM, did not affect bacterial or fungal gamma diversity (Fig. S7, Table S3). These relationships were robust to the use of different richness estimators (Table S3).

Soil bacteria and fungi, however, exhibited markedly different diversity patterns within islands. Bacterial alpha diversity, measured as the average OTU richness per sample within an island, increased with island area, whereas fungal alpha diversity was unaffected by island area (Fig. 2c, d). By contrast, bacterial beta diversity, measured as the average Bray-Curtis dissimilarity among samples within an island, was not related to island area, whereas fungal beta diversity significantly increased with area (Fig. 2e, f and Fig. S8). Qualitatively similar results were obtained when only considering beta diversity among samples within the 20 m × 20 m plot (Fig. S9). These results suggest that the assembly of bacterial and fungal communities was likely driven by different mechanisms. Neither bacterial nor fungal within-island (alpha and beta) diversity was affected by island isolation (Fig. S7, Table S3).

**Fig. 3:**
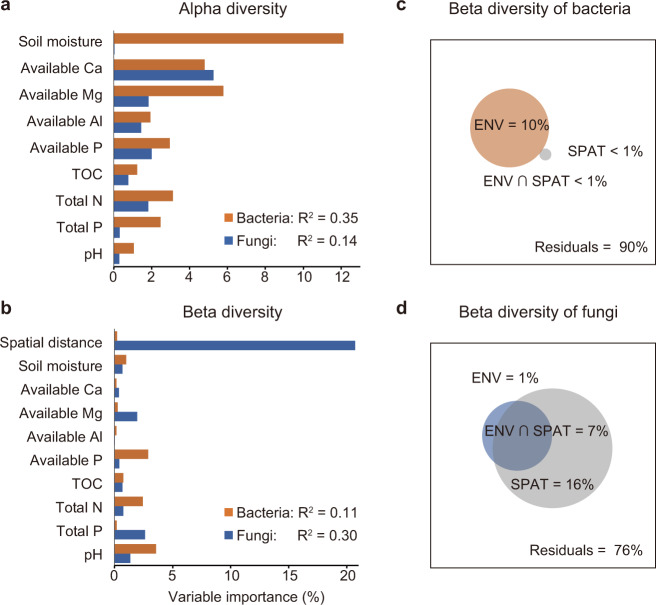
The fraction of the variation in alpha and beta diversity of soil bacteria and fungi explained by environmental and spatial predictors. The importance of individual predictor variables was assessed using multiple linear regression models of alpha (**a**) and beta (**b**) diversity, and the relative importance of soil environment variables (ENV) and spatial distance (SPAT) among samples within island was assessed using variation partitioning of bacterial (**c**) and fungal (**d**) beta diversity.

We tested the roles of environmental filtering and spatial processes on soil microbial assembly on the TIL islands, by comparing the variation in within-island alpha and beta diversity explained by environmental and spatial variables. We found that soil moisture, as well as the habitat heterogeneity of all soil properties, significantly increased with island area (Table S4). All other soil properties were not related to island area (Table S4). Multiple regression showed that soil properties accounted for a sizable fraction (~35%) of the variation in bacterial alpha diversity. Soil moisture was identified as the most important predictor of bacterial alpha diversity, explaining more than 12% of the variation alone (Fig. 3a). In contrast, soil properties accounted for much less variation in fungal alpha diversity. Soil available Ca, the best explanatory variable, explained <6% of the variation in fungal alpha diversity; each of the other soil properties accounted for less than 2% (Fig. 3a). Multiple regressions on distance matrices showed that while a small yet significant portion (~11%) of the variation in bacterial beta diversity can be explained by the dissimilarities in soil properties among samples, virtually none can be explained by spatial distance among samples (Fig. 3b). In contrast, spatial distances among samples were a substantially better predictor of fungal beta diversity than soil properties (Fig. 3b). These results were further confirmed by variation partitioning of beta diversity into environmental and spatial components, which showed that environmental and spatial variables explained a substantially larger fraction of variation in bacterial and fungal beta diversity, respectively (Fig. 3c, d).

**Fig. 4:**
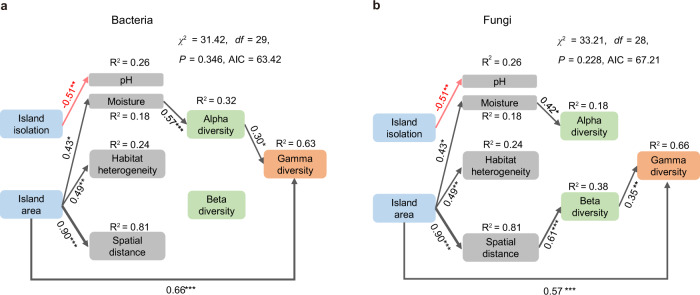
Structural equation models considering the direct and indirect pathways via which island area and isolation influence alpha, beta, and gamma diversity of soil bacteria and fungi. Panel **a** is for bacteria; panel **b** is for fungi. Island isolation was measured as distance to the nearest island (note that using the nearest distance to the mainland yielded similar results). Black and red arrows indicate significant positive and negative pathways, respectively (*P < 0.05, **P < 0.01, ***P < 0.001). Numbers along the arrows indicate standardized path coefficients. *R*^2^ represents the proportion of variance explained for the dependent variable.

We further used SEM to link microbial assembly mechanisms to island-scale species-area relationships. SEM revealed that besides the direct link between island area and gamma diversity, which is indicative of the sampling effect, greater bacterial and fungal gamma diversity on larger islands were associated with greater alpha diversity within samples and greater beta diversity between samples, respectively (Fig. 4a, b). For bacteria, the greater alpha diversity on larger islands was driven by higher soil moisture on those islands; habitat heterogeneity, which was higher on larger islands, as well as spatial distance among samples, had little effect on bacterial alpha or beta diversity (Fig. 4a). For fungi, the greater beta diversity on larger islands was mainly driven by the increased spatial distance among samples; habitat heterogeneity also did not affect fungal beta diversity (Fig. 4b).

## Discussion

Our study provides, to our knowledge, the first empirical evidence that soil bacteria and fungi on islands exhibited similar island-scale biogeographic patterns, but that these similar patterns were driven by strikingly different mechanisms. For soil bacteria, the loss of island-scale diversity on smaller islands was mainly caused by the decline of within-sample (alpha) diversity within island. At first glance, this pattern appears to be in line with the prediction of the area *per se* effect (see Fig. 1b), which suggests that higher alpha diversity on larger islands can be driven by a neutral equilibrium between species immigration and extinction, as postulated by the theory of island biogeography [4]. However, we found that island area influenced alpha diversity mainly through altering soil properties, particularly soil moisture (Figs. 3 and 4), pointing to the importance of variation in habitat quality among islands for driving the observed bacterial species-area relationship (Fig. 1b). This aspect of our findings is thus consistent with previous work showing that bacteria tend to be more responsive than fungi to water stress [53–55]. In our study, smaller islands are characterized by lower vegetation density, as well as by increased edge effects associated with their greater island perimeter to area ratio [36], which may have led to increased insolation, wind exposure and evapotranspiration, and, in turn, lower soil moisture. Overall, our results illustrate habitat quality as an important determinant of soil bacterial diversity. Likewise, several other studies have found that environmental variables better predict soil bacterial community structure than spatial distance across various spatial scales (e.g., [12, 56, 57]).

Unlike bacteria, the positive island species-area relationship for fungi was primarily driven by increased beta diversity among localities on larger islands (Fig. 4b). The increased beta diversity has typically been interpreted as evidence for greater habitat heterogeneity on larger islands [28, 32, 58]. Neutral theory, however, suggests that the same pattern could arise from dispersal limitation [35, 59], without incurring differences in species or environmental characteristics. In our study, although the heterogeneity of soil properties increased on larger islands, habitat heterogeneity was not a significant predictor of fungal beta diversity within islands. Instead, spatial distance between samples strongly influenced fungal within-island beta diversity. These results thus point to the importance of dispersal limitation in shaping fungal community assembly within islands, and, in turn, the observed fungal species-area relationship (Fig. 1c). Note that under dispersal limitation, the presence of a tradeoff between species dispersal and competitive ability may also contribute to increased fungal beta and gamma diversity for larger islands [60], a mechanism that cannot be evaluated with our current data. Fungal communities are known to be more influenced by dispersal limitation than bacteria [61, 62]. Recent research has shown that the majority of fungal spores disperse over short distances (from centimeters to meters; [63]), and that dispersal limitation can operate to influence fungal communities within a scale of less than 1 km [21, 64]. In our study system, although aeolian processes may transport spores of various fungal species among islands, with island isolation seemingly posing little barrier for dispersal (see next paragraph), the presence of forest canopy on islands may lower wind velocity and reduce the dispersal of fungal spores within island [65]. Such dispersal limitation is likely stronger on larger islands, which support more dense forest canopy [66]. In line with this prediction, we found that fungal beta diversity among samples within a 20 m × 20 m plot also significantly increased with island area, even though these samples share similar spatial distances across all islands (Fig. S9). In contrast, island area and spatial distance were poor predictors of bacterial beta diversity within islands and plots (Figs. 3 and 4, and Fig. S9).

It is notable that for both bacteria and fungi, their island-level (gamma) diversity was not affected by island isolation, a pattern at odds with the theory of island biogeography predicting that more isolated islands should contain fewer species [4]. Similar non-significant species-isolation relationships have also been found for higher organisms (e.g., plants, birds, lizards, small mammals) inhabiting the TIL islands [36, 66, 67]. Together, these results suggest that the spatial discreteness of the TIL islands did not result in across-island dispersal limitation for a variety of organisms, probably due to the relatively recent isolation history (~60 years) of these islands and their close proximity to the mainland (<4 km).

Several limitations of our study are worth noting. First, DNA markers used for bacteria and fungi (i.e., 16 S for bacteria and ITS2 for fungi) are known to have different taxonomic resolutions [68]. It is therefore possible that bacterial communities may exhibit finer spatial structure that cannot be detected with current methods, and that fungal communities may exhibit environmental signatures that are visible only at coarser taxonomic levels [69]. Future studies should explore these possibilities. Second, given the presence of a large number of rare microbial species and the difficulty of capturing them during sampling and sequencing, the accuracy of estimated microbial diversity values may be questioned. The consistency of our results based on different diversity metrics eases this concern (Tables S2, S3), although the robustness of these metrics in the context of evaluating species-area mechanisms still needs to be investigated further. Third, despite the measurement of common soil environmental variables, it is worth noting that other unmeasured variables could also influence microbial communities. It is possible that these unmeasured variables, including plant community structure and historical factors (e.g., past dispersal events, past environmental conditions), could be spatially structured and influence microbial communities through the apparent effect of spatial distance [70]. Therefore, we cannot completely exclude the role of habitat heterogeneity in contributing to fungal species-area relationships. Nevertheless, analyses of our microbial data using null models, without considering soil environmental variables, also indicate that dispersal limitation dominated the assembly of soil fungal communities within the TIL islands (see [71]).

While studies of habitat loss and fragmentation in the Thousand-Island Lake and elsewhere have traditionally focused on their effects on higher organisms [36, 72, 73], our study revealed that both bacterial and fungal diversity declined on smaller islands, providing direct empirical evidence that habitat loss and fragmentation are also threatening the diversity of microorganisms. Our study further reveals that the loss of bacterial and fungal diversity on smaller islands is driven by contrasting mechanisms. Poorer habitat quality on smaller islands drives the systematic decline of diversity within samples for bacteria, whereas reduced dispersal limitation on smaller islands causes lower diversity among samples for fungi. These contrasting assembly mechanisms defy a universal explanation for the similar island biogeographic patterns observed for bacteria and fungi, with important implications for the conservation of bacterial and fungal diversity. In particular, the different mechanisms suggest that preventing habitat degradation would be an effective strategy for reducing the extinction risk of soil bacteria, whereas maintaining large habitat fragments would be more effective for the conservation of soil fungal diversity. The generality of our results, however, would need to be assessed for other island systems.

## Supplementary information

Supplementary Information

## Data Availability

DNA sequence data are accessible at the NCBI-SRA under the accession number PRJNA517449. All other data that support the findings of this study can be accessed via the Dryad Digital Repository (10.5061/dryad.gqnk98sj8).
