# Supplementary material for: Island biogeography of soil bacteria and fungi: similar patterns, but different mechanisms
Source: ISME J. 2020 Apr 27;14(7):1886–96. doi: 10.1038/s41396-020-0657-8 (PMC7305213; doi:10.1038/s41396-020-0657-8)
Supplement: Supplementary file 1 — Supplementary Information [file 41396_2020_657_MOESM1_ESM.pdf]

Supplementary Information for

Island biogeography of soil bacteria and fungi: similar patterns, but different mechanisms

Shao-peng Li[1,2,3], Pandeng Wang[1,4], Yongjian Chen[4], Maxwell C. Wilson[5], Xian Yang[1], Chao Ma[6], Jianbo Lu[7], Xiao-yong Chen[2,8], Jianguo Wu[5], Wen-sheng Shu[9,]* & Lin Jiang[1,]*

***Correspondence to:**

Lin Jiang (lin.jiang@biology.gatech.edu) or Wen-sheng Shu (shuws@mail.sysu.edu.cn)

This PDF file includes:

Figure S1 to S9

Table S1 to S4

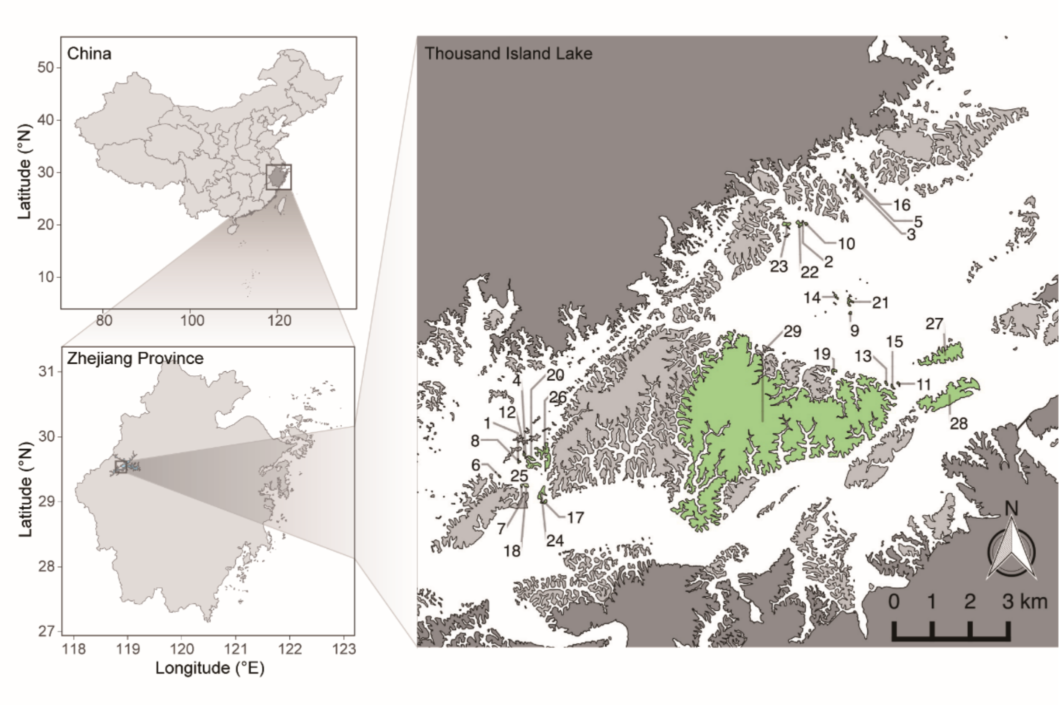

Figure S1. The locations of the 29 study islands in the Thousand Island Lake, China. The sampled islands are colored in green and ranked according to their area (from smallest to largest).

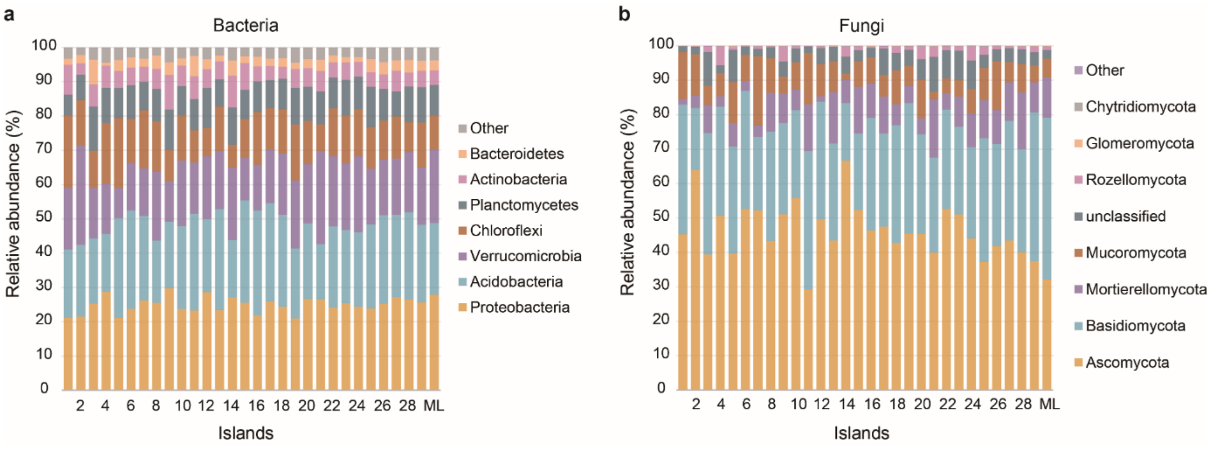

Figure S2. The relative abundance of dominant lineages (at the phylum level) on each island and the nearby mainland (ML). The sampled islands are ranked according to their area (from smallest to largest).

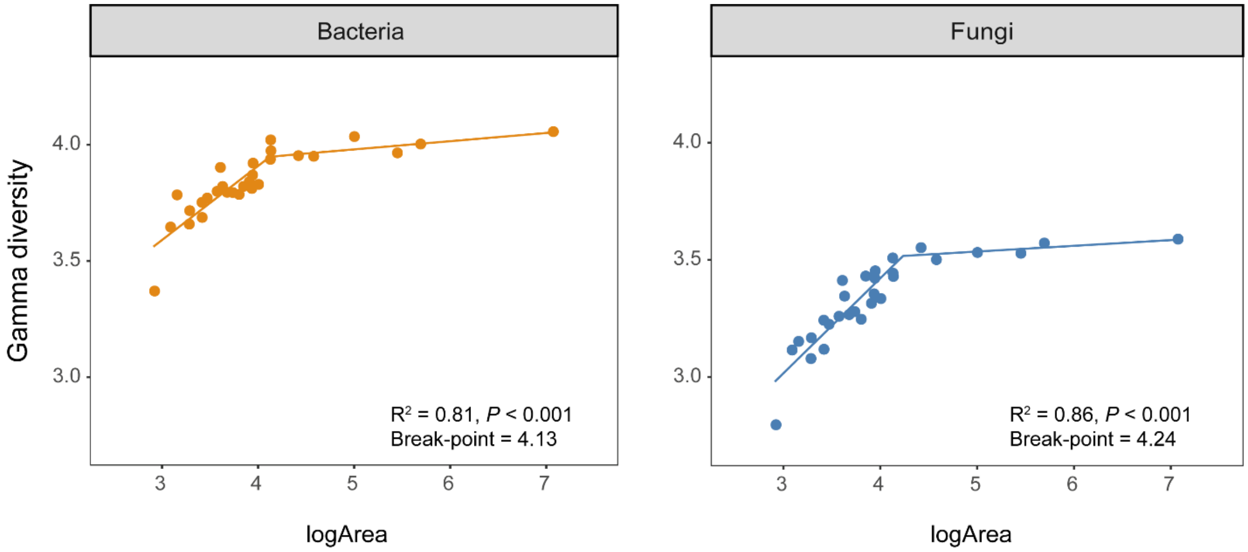

Figure. S3. Segmented regressions showing the breaking points for the effects of island area on the gamma diversity of bacteria and fungi. Gamma diversity-area relationships can be better modeled by segmented regressions than simple linear regression according to the Akaike information criterion (AIC) values, indicating the existence of island threshold size (~2 ha) below which diversity precipitously declined.

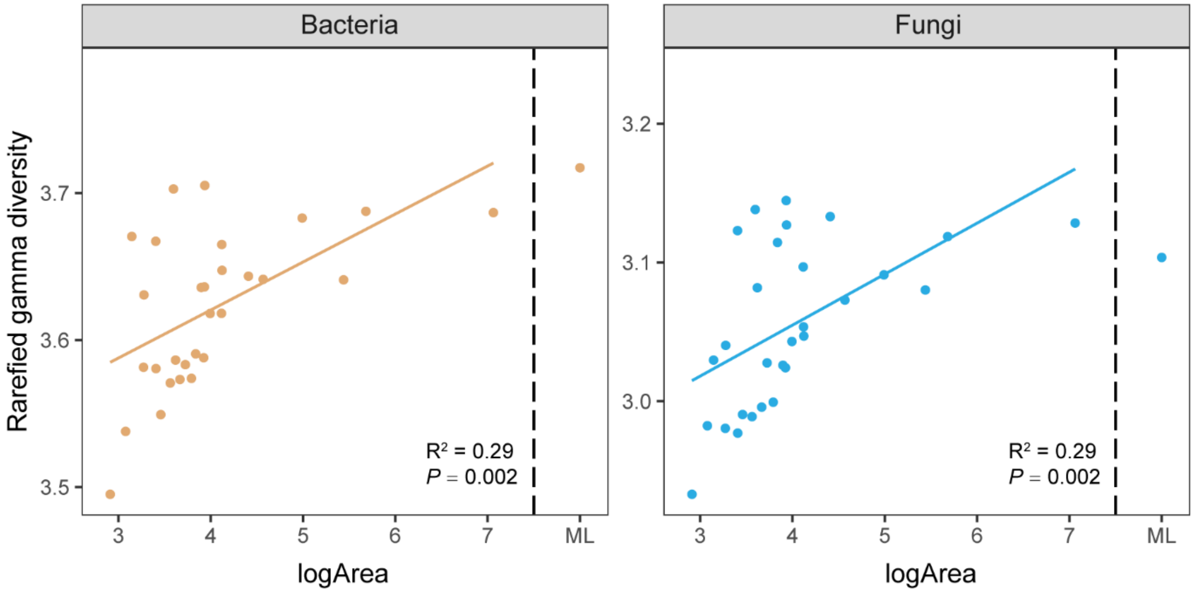

Figure S4. The effect of island area on the rarefied gamma diversity of soil bacteria and fungi. Gamma diversity was rarefied to the same number of reads (16,762 and 5,580 sequences for bacteria and fungi, respectively) for each island. Rarefied gamma diversity still showed significant positive relationships with island area for both bacteria and fungi, indicating that the sampling effect is not the sole mechanism responsible for the observed island species-area relationships. The rarefication was run 999 times. ML indicates mainland.

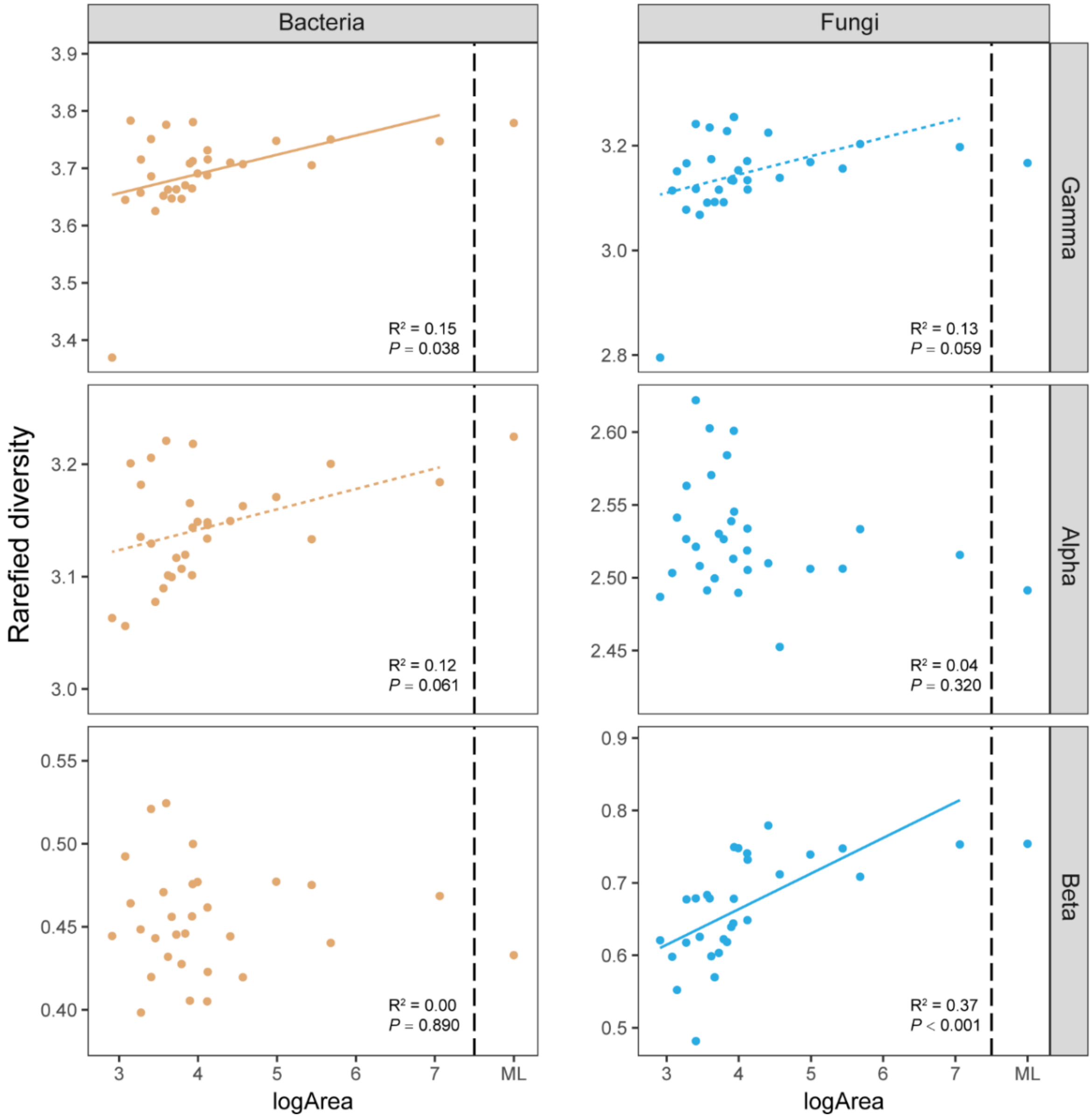

Figure S5. The effect of island area on the rarefied gamma, alpha and beta diversity, when all islands were rarefied to equal sample size. We calculated the three diversity components by rarifying down to four samples per island (besides the smallest island with two samples). The solid lines represent significant linear regressions ($P < 0.05$), and the dashed lines represent marginally significant regressions ($P < 0.10$). ML indicates mainland.

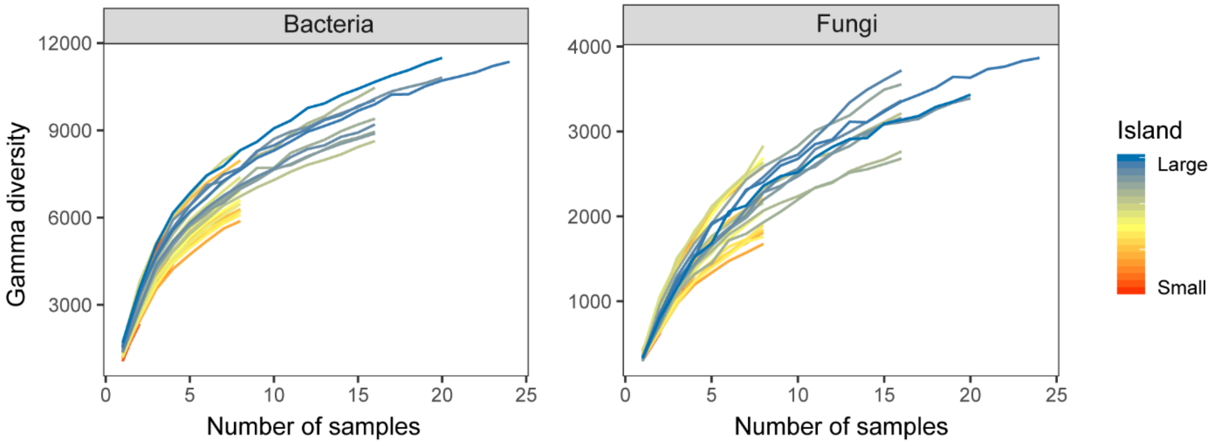

Figure S6. The relationship between the estimated gamma diversity and the number of soil samples on the 29 islands. All islands shared similar rarefaction curves, and gamma diversity were generally higher on larger islands at equal sample size. The sampled islands are colored according to their area.

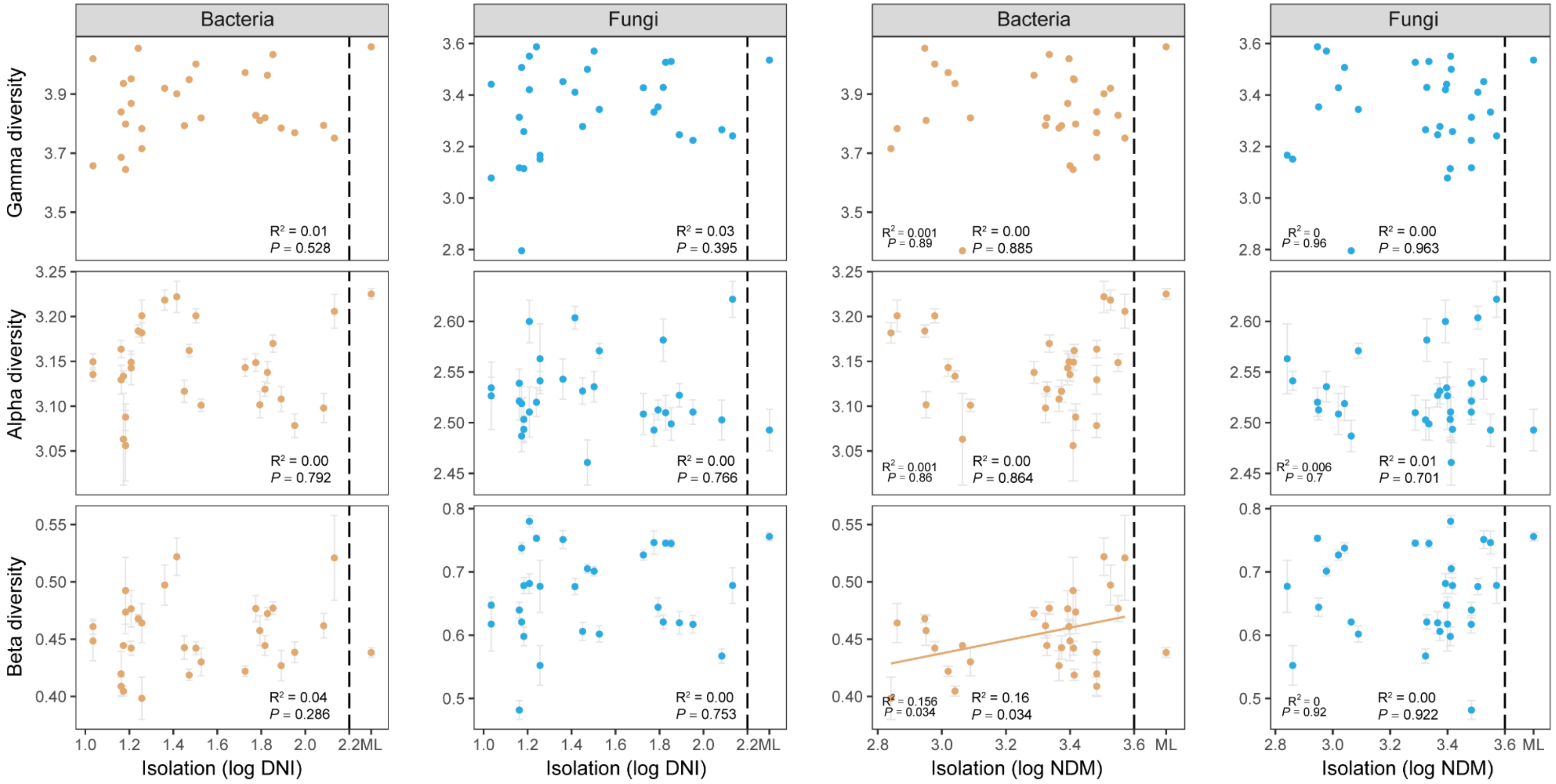

Figure S7. The effect of island isolation on the alpha, beta and gamma diversity of soil bacteria and fungi. Gamma diversity was measured as the estimated total OTU richness (based on Chao2 estimator) per island. Alpha diversity was measured as the average OTU richness per sample within each island. Beta diversity was measured as average pairwise Bray-Curtis dissimilarities among samples within each island. Error bars represent standard error. Island isolation was measured as distance to the nearest island (DNI) and the nearest distance to the mainland (NDM). ML indicates mainland.

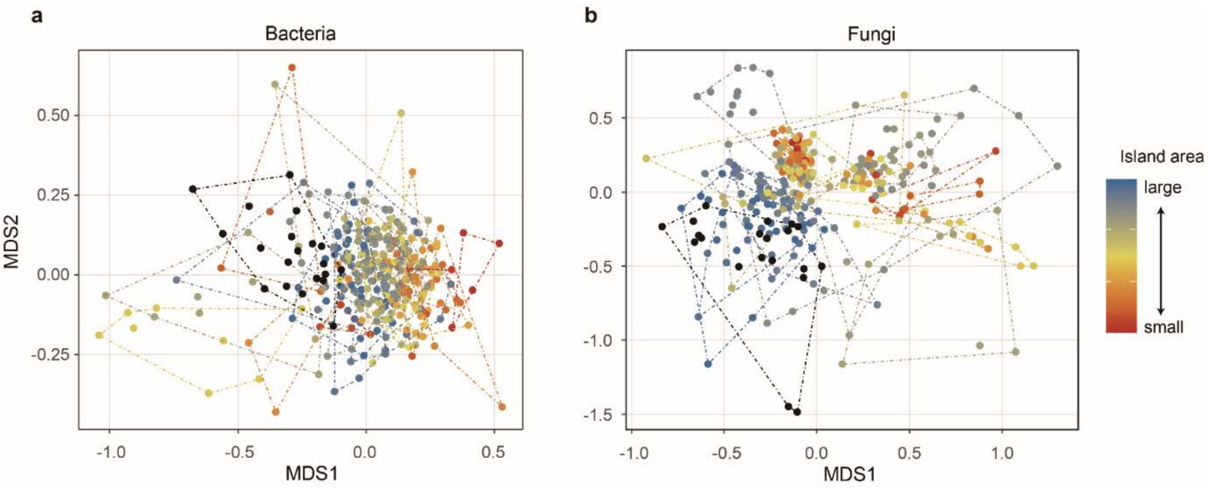

Figure S8. Ordination of the 306 samples by two-dimensional nonmetric multidimensional scaling (NMDS). NMDS was based on pairwise Bray–Curtis dissimilarity values. Samples are colored according to island size, with black dots representing the samples from the mainland.

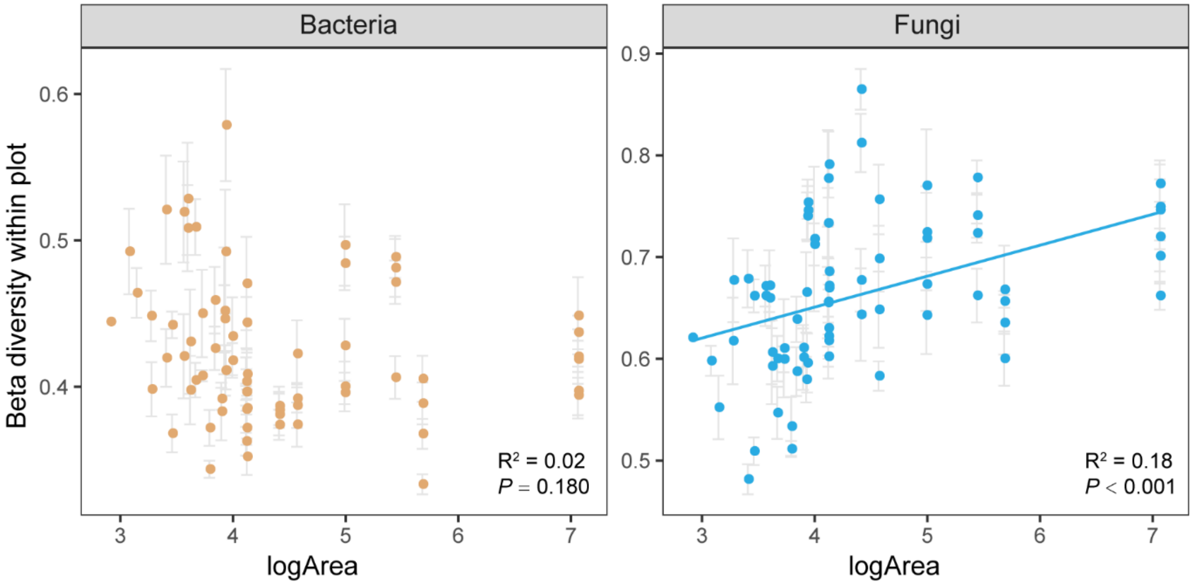

Figure S9. The effect of island area on the beta diversity within plot for soil bacteria and fungi. Beta diversity was measured as average pairwise Bray-Curtis dissimilarities among four samples within the same plot (20 m × 20 m) on each island. Error bars represent standard error.

Table S1. Soil properties of the 29 study islands and the nearby mainland sites (ML) in the Thousand Island Lake region, China. Each mean in table has an associated SE.

Island	Soil moisture (%)	Available Ca (mg/kg)	Available Mg (mg/kg)	Available Al (mg/g)	Available P (mg/kg)	TOC (%)	Total N (mg/g)	Total P (mg/g)	pH
1	13.36±0.47	58.4±15.9	25.3±8.8	1.14±0.13	3.21±0.19	18.41±0.24	1.37±0.10	0.28±0.00	4.36±0.07
2	9.34±1.23	60.2±12.8	13.6±1.8	1.45±0.20	1.82±0.22	13.88±0.71	0.88±0.05	0.16±0.01	4.09±0.06
3	13.99±1.04	282.9±43.9	39.6±6.1	1.27±0.07	3.19±0.64	15.83±2.58	1.28±0.20	0.30±0.05	4.30±0.05
4	11.14±1.85	60.9±6.3	23.5±2.1	1.34±0.15	2.95±0.19	22.21±1.89	1.37±0.07	0.21±0.02	4.24±0.02
5	14.66±1.15	223.7±51.8	31.9±4.7	1.20±0.16	5.05±0.37	26.86±1.35	1.86±0.08	0.28±0.02	4.15±0.07
6	14.09±1.55	286.5±66.2	40.8±7.3	1.21±0.08	7.27±1.31	27.35±3.80	1.90±0.28	0.19±0.03	4.03±0.05
7	12.20±0.67	43.3±7.3	24.3±3.4	0.93±0.15	2.60±0.17	15.84±3.13	1.10±0.12	0.19±0.01	4.37±0.04
8	10.46±0.95	54.9±9.7	12.7±1.4	1.08±0.13	2.65±0.17	26.51±2.30	1.44±0.10	0.15±0.02	4.12±0.03
9	9.66±0.55	59.6±11.0	20.5±3.5	0.95±0.13	2.76±0.19	12.13±1.20	1.00±0.05	0.28±0.01	4.35±0.04
10	18.28±0.53	1153.0±196.5	158.4±20.2	1.13±0.09	6.88±0.39	22.04±2.31	1.44±0.15	0.13±0.01	4.43±0.08
11	14.02±0.64	79.8±9.1	28.0±3.8	1.13±0.08	3.38±0.21	23.10±2.27	1.42±0.11	0.27±0.01	4.17±0.06
12	13.75±0.88	108.2±23.1	18.7±2.0	0.76±0.08	5.16±1.39	29.09±2.58	1.50±0.17	0.32±0.01	4.16±0.05
13	9.42±0.26	50.1±6.1	14.0±1.0	0.96±0.05	3.13±0.10	18.74±2.14	1.14±0.08	0.20±0.02	4.21±0.02
14	16.33±1.37	63.5±10.9	16.9±2.2	0.81±0.08	3.11±0.23	18.18±1.76	1.26±0.08	0.30±0.02	4.14±0.08
15	16.14±0.85	118.5±16.3	19.5±1.4	1.31±0.05	3.69±0.27	22.76±2.59	1.41±0.11	0.25±0.01	4.09±0.03
16	14.50±1.07	62.2±12.3	23.6±4.7	0.83±0.11	3.51±0.31	19.05±1.81	1.30±0.11	0.22±0.02	4.30±0.04
17	16.19±0.55	56.2±12.6	20.9±3.1	1.31±0.12	2.79±0.17	21.87±1.56	1.40±0.07	0.24±0.03	4.17±0.04
18	18.80±1.04	90.0±19.0	22.4±3.3	1.12±0.12	3.23±0.18	32.52±2.86	1.83±0.07	0.26±0.02	4.13±0.03
19	16.13±0.56	831.5±114.0	82.9±7.3	1.19±0.12	5.57±0.72	17.48±0.89	1.07±0.05	0.10±0.00	4.48±0.07
20	12.29±1.03	87.6±13.3	24.8±4.3	1.01±0.03	3.14±0.16	28.30±1.27	1.75±0.05	0.29±0.02	4.03±0.10
21	13.13±0.56	71.1±8.7	34.7±5.2	1.08±0.05	3.95±0.19	18.25±1.01	1.31±0.04	0.35±0.02	4.30±0.04
22	13.71±0.81	72.6±13.8	60.9±17.1	1.08±0.08	2.88±0.11	20.11±2.00	1.33±0.08	0.18±0.02	4.39±0.06
23	13.67±0.45	64.3±7.8	24.0±2.5	1.02±0.05	2.90±0.10	22.82±1.25	1.40±0.06	0.26±0.01	4.34±0.04
24	14.34±0.56	73.1±8.8	31.5±3.2	1.12±0.08	3.26±0.17	31.17±2.48	1.88±0.13	0.29±0.03	4.26±0.04
25	15.70±0.49	41.2±4.5	19.3±1.5	1.01±0.08	2.88±0.10	17.94±1.61	1.20±0.06	0.15±0.01	4.20±0.03
26	14.67±0.71	67.6±12.9	33.5±6.1	0.97±0.04	2.90±0.27	20.42±1.60	1.41±0.10	0.21±0.02	4.29±0.04
27	14.61±0.74	68.5±14.9	18.6±2.6	0.84±0.06	2.66±0.18	23.96±2.74	1.41±0.13	0.25±0.01	4.18±0.03
28	17.42±0.70	64.7±7.9	22.4±2.5	0.98±0.08	3.18±0.18	27.73±1.24	1.68±0.07	0.24±0.01	4.10±0.04
29	16.76±0.94	71.9±7.5	27.9±4.2	1.13±0.06	3.21±0.12	27.30±1.95	1.60±0.10	0.22±0.02	4.20±0.04
ML	15.98±0.43	136.4±11.2	59.8±5.1	1.00±0.04	2.88±0.12	29.10±1.49	1.73±0.07	0.21±0.01	4.37±0.02

Table S2. Characteristics and microbial diversity of the 29 study islands and the nearby mainland sites (ML) in the Thousand Island Lake region, China. Refer to Fig. S1 for survey sites.

Island	Latitude (N)	Longitude (E)	Area (m^2)	NDM (m)	DNI (m)	Bacteria Gamma	Fungi Gamma	Bacteria Alpha	Fungi Alpha	Bacteria Beta	Fungi Beta
1	29°34'9"	118°53'56"	815	1160	15	2342	624	1165±137	307±11	0.44	0.62
2	29°30'52"	118°49'14"	1197	2567	15	4414	1301	1152±106	320±15	0.49±0.03	0.60±0.01
3	29°34'50"	118°54'38"	1396	725	18	6070	1416	1592±64	348±7	0.46±0.02	0.55±0.03
4	29°30'53"	118°49'22"	1870	2511	11	4543	1196	1366±23	339±24	0.45±0.02	0.62±0.04
5	29°34'48"	118°54'37"	1890	694	18	5192	1466	1521±39	369±27	0.40±0.02	0.68±0.04
6	29°32'50"	118°54'41"	2538	3725	136	5636	1743	1611±69	420±18	0.52±0.04	0.68±0.03
7	29°30'29"	118°49'17"	2550	3044	15	4853	1310	1350±51	333±15	0.42±0.02	0.48±0.01
8	29°30'38"	118°48'56"	2878	3040	90	5881	1675	1202±37	325±9	0.44±0.01	0.62±0.01
9	29°30'49"	118°49'15"	3645	2615	15	6286	1812	1229±42	313±9	0.47±0.02	0.68±0.01
10	29°33'4"	118°54'28"	3949	3205	26	7965	2576	1677±67	402±11	0.52±0.02	0.68±0.01
11	29°34'7"	118°53'60"	4160	1228	34	6599	2209	1263±20	373±6	0.43±0.01	0.60±0.01
12	29°31'50"	118°55'27"	4638	2106	121	6225	1842	1259±46	321±14	0.46±0.01	0.57±0.01
13	29°30'56"	118°49'20"	5307	2363	28	6213	1896	1312±38	341±10	0.44±0.01	0.61±0.01
14	29°31'51"	118°55'15"	6181	2321	78	6094	1760	1287±40	337±9	0.43±0.01	0.62±0.02
15	29°31'47"	118°55'21"	6876	2128	66	6604	2688	1317±25	385±18	0.44±0.01	0.62±0.01
16	29°30'29"	118°49'22"	7880	3043	15	6913	2059	1460±32	347±11	0.41±0.01	0.64±0.01
17	29°34'46"	118°54'46"	8388	893	62	6467	2258	1269±41	326±7	0.46±0.01	0.64±0.01
18	29°30'16"	118°49'41"	8542	2471	16	7389	2635	1398±57	401±20	0.48±0.02	0.68±0.02
19	29°33'0"	118°54'40"	8625	3359	23	8310	2831	1657±42	352±16	0.50±0.02	0.75±0.01
20	29°32'1"	118°54'23"	9856	3547	60	6732	2156	1410±32	312±11	0.48±0.01	0.75±0.02
21	29°34'6"	118°53'54"	13071	1099	15	8629	3213	1362±19	334±12	0.40±0.00	0.74±0.01
22	29°30'52"	118°49'26"	13172	2494	11	10464	2765	1415±29	344±8	0.46±0.01	0.65±0.00
23	29°34'6"	118°53'39"	13264	1046	53	9397	2681	1395±31	328±14	0.42±0.00	0.73±0.01
24	29°30'22"	118°49'37"	25619	2576	16	8950	3555	1414±29	332±19	0.44±0.01	0.78±0.01
25	29°30'46"	118°49'30"	36958	2588	30	8894	3161	1455±23	295±14	0.42±0.00	0.71±0.01
26	29°30'59"	118°49'39"	97930	2164	71	10813	3390	1486±34	319±11	0.48±0.01	0.74±0.01
27	29°32'10"	118°55'56"	274942	1939	67	9200	3364	1381±38	327±13	0.47±0.01	0.75±0.01
28	29°31'30"	118°55'52"	479759	950	32	10043	3719	1592±29	346±12	0.44±0.01	0.70±0.01
29	29°31'55"	118°53'22"	11538756	884	17	11354	3866	1532±26	335±11	0.47±0.00	0.75±0.00
ML	29°39'10"	119°0'27"				11489	3434	1682±23	318±15	0.44±0.00	0.76±0.01

* The sampled islands are ranked according to their area (from smallest to largest). Island isolation was measured as the nearest distance to the mainland (NDM) and distance to the nearest island (DNI). Gamma diversity was measured as the estimated total OTU richness (based on Chao2 estimator) per island. Alpha diversity was measured as the average OTU richness per sample within each island. Beta diversity was measured as average pairwise Bray-Curtis dissimilarities among samples within each island. Each mean in table has an associated SE.

Table. S3 The effect of island area and isolation on the alpha, beta and gamma diversity of soil bacteria and fungi.

Independent Variable	Dependent variable		Bacteria			Fungi		
			Slope	R^2	*P*	Slope	R^2	*P*
Island area	Gamma diversity	Chao1	**0.108**	**0.60**	**0.000**	**0.131**	**0.61**	**0.000**
		Chao2	**0.124**	**0.56**	**0.000**	**0.159**	**0.59**	**0.000**
		Jack1	**0.129**	**0.61**	**0.000**	**0.151**	**0.62**	**0.000**
		Jack2	**0.133**	**0.58**	**0.000**	**0.162**	**0.60**	**0.000**
		ACE	**0.106**	**0.61**	**0.000**	**0.134**	**0.63**	**0.000**
		ICE	**0.123**	**0.54**	**0.000**	**0.165**	**0.58**	**0.000**
	Alpha diversity	OTU richness (q = 0)	0.018	0.13	0.058	-0.008	0.03	0.352
		Exponential of Shannon entropy (q = 1)	0.042	0.10	0.097	**-0.042**	**0.14**	**0.047**
		Inverse Simpson (q =2)	0.049	0.09	0.119	**-0.051**	**0.16**	**0.035**
	Beta diversity	Jaccard	0.004	0.09	0.108	**0.022**	**0.22**	**0.010**
		Bray-Curtis	0.001	0.00	0.903	**0.049**	**0.36**	**0.001**
		RC_{bray}	0.157	0.10	0.101	0.105	0.13	0.054
		1-CqN (q = 0)	0.005	0.09	0.110	**0.027**	**0.22**	**0.011**
		1-CqN (q = 1)	0.003	0.01	0.649	**0.058**	**0.35**	**0.001**
		1-CqN (q = 2)	-0.016	0.04	0.274	**0.069**	**0.24**	**0.006**
Island isolation (DNI)	Gamma diversity	Chao1	0.046	0.02	0.517	0.075	0.03	0.376
		Chao2	0.053	0.01	0.528	0.089	0.03	0.395
		Jack1	0.053	0.01	0.532	0.074	0.02	0.452
		Jack2	0.062	0.02	0.489	0.086	0.02	0.415
		ACE	0.046	0.02	0.502	0.065	0.02	0.443
		ICE	0.060	0.02	0.481	0.094	0.03	0.391
	Alpha diversity	OTU richness (q = 0)	-0.007	0.00	0.792	0.006	0.00	0.766
		Exponential of Shannon entropy (q = 1)	0.035	0.01	0.600	-0.028	0.01	0.627
		Inverse Simpson (q =2)	0.101	0.05	0.227	-0.025	0.01	0.704
	Beta diversity	Jaccard	-0.002	0.00	0.776	0.000	0.00	0.989
		Bray-Curtis	0.020	0.04	0.286	0.013	0.00	0.753
		RC_{bray}	0.154	0.01	0.550	0.048	0.00	0.748
		1-CqN (q = 0)	-0.002	0.00	0.764	-0.002	0.00	0.957
		1-CqN (q = 1)	0.014	0.02	0.427	0.001	0.00	0.977
		1-CqN (q = 2)	0.055	0.08	0.143	0.016	0.00	0.828

	Gamma diversity	Chao1	-0.035	0.00	0.741	-0.036	0.00	0.778
		Chao2	-0.018	0.00	0.885	-0.007	0.00	0.963
		Jack1	-0.022	0.00	0.861	-0.024	0.00	0.867
		Jack2	-0.011	0.00	0.937	-0.013	0.00	0.937
		ACE	-0.028	0.00	0.786	-0.029	0.00	0.820
		ICE	-0.005	0.00	0.970	0.000	0.00	0.998
Island isolation (NDM)	Alpha diversity	OTU richness (q = 0)	-0.007	0.00	0.864	0.012	0.01	0.701
		Exponential of Shannon entropy (q = 1)	0.002	0.00	0.987	0.129	0.08	0.126
		Inverse Simpson (q =2)	0.003	0.00	0.978	0.120	0.06	0.214
	Beta diversity	Jaccard	0.001	0.00	0.882	-0.015	0.01	0.685
		Bray-Curtis	**0.056**	**0.16**	**0.034**	-0.006	0.00	0.922
		RC_{bray}	0.299	0.02	0.431	-0.081	0.00	0.711
		1-CqN (q = 0)	0.002	0.00	0.857	-0.015	0.00	0.737
		1-CqN (q = 1)	0.051	0.13	0.050	-0.015	0.00	0.842
		1-CqN (q = 2)	**0.157**	**0.29**	**0.002**	0.046	0.01	0.666

* Gamma diversity was calculated as the total OTU richness per island based on Chao1, Chao2, Jack1, Jack2, ACE, and ICE estimators. Alpha diversity was measured as average OTU richness, the exponent of Shannon entropy, and the inverse Simpson index per sample, which are equal to the effective number of species based on Hill numbers (q = 0, 1, 2, respectively). Beta diversity was measured as average Jaccard, Bray-Curtis dissimilarity, and the Bray–Curtis based Raup–Crick (RC_{bray}) values among samples within each island, as well as the unifying similarity and differentiation measures through Hill numbers (1-CqN, q = 0, 1, 2). RC_{bray} was generated by a statistical null modeling approach following the procedure described by Stegen et al. (2015). Diversity measures through Hill numbers were calculated following Chao et al. (2014). Island isolation was measured as distance to the nearest island (DNI) and nearest distance to the mainland (NDM). Significant values are highlighted in bold ($P < 0.05$).

Chao, A., Chiu, C-H., Jost, L. Unifying species diversity, phylogenetic diversity, functional diversity, and related similarity and differentiation measures through Hill numbers. *Annu. Rev. Ecol. Syst*. **45**, 297-324 (2014).

Stegen, J.C., Lin, X.J., Fredrickson, J.K., Konopka, A.E. Estimating and mapping ecological processes influencing microbial community assembly. *Front. Microbiol*. **6**, 370 (2015).

Table S4. The influences of island area and isolation on soil properties and habitat heterogeneity at the island level. Significant values are highlighted in bold (P < 0.05). Soil properties were standardized to have a mean value of 0 and variance of 1.

Independent Variable	Dependent Variable	Slope	R^2	*P*
Island area	**Soil moisture**	**0.361**	**0.18**	**0.020**
	Available Ca	-0.242	0.05	0.244
	Available Mg	-0.075	0.01	0.647
	Available Al	-0.183	0.08	0.143
	Available P	-0.132	0.02	0.522
	TOC	0.237	0.11	0.083
	Total N	0.182	0.06	0.202
	Total P	-0.016	0.00	0.924
	pH	-0.062	0.01	0.662
	**Habitat heterogeneity**	**0.045**	**0.24**	**0.007**
Island isolation (DNI)	Soil moisture	0.276	0.02	0.518
	Available Ca	0.112	0.00	0.839
	Available Mg	-0.432	0.04	0.313
	Available Al	-0.469	0.07	0.153
	Available P	0.542	0.04	0.313
	TOC	0.645	0.11	0.073
	Total N	0.505	0.07	0.178
	Total P	0.136	0.00	0.760
	**pH**	**-0.977**	**0.26**	**0.005**
	Habitat heterogeneity	0.058	0.06	0.214
Island isolation (NDM)	Soil moisture	-0.693	0.05	0.269
	Available Ca	0.322	0.01	0.693
	Available Mg	0.209	0.00	0.743
	Available Al	-0.509	0.04	0.299
	Available P	0.394	0.01	0.622
	TOC	-0.142	0.00	0.795
	Total N	-0.468	0.03	0.403
	**Total P**	**-1.704**	**0.25**	**0.006**
	pH	0.078	0.00	0.888
	Habitat heterogeneity	0.011	0.00	0.879
